# Epidemiological and clinical aspects of psychiatric disorders in Tunisian prisons

**DOI:** 10.1192/j.eurpsy.2022.1551

**Published:** 2022-09-01

**Authors:** M. Kacem, W. Bouali, N. Karkeni, N. Faouel, L. Zarrouk

**Affiliations:** University Hospital of Mahdia, Tunisia., Psychiatry, mahdia, Tunisia

**Keywords:** Prevalence, Psychiatric Disorder, Prison, penitentiary psychiatry

## Abstract

**Introduction:**

The estimation of the percentage of mentally ill among prisoners and of the most severe psychiatric disorders has been the subject of few studies in Tunisia.

**Objectives:**

To study in a general way the extent of psychiatric disorders among prisoners. To describe the socio-demographic and judicial characteristics of prisoners. To describe the clinical, evolutionary and therapeutic characteristics of the main psychiatric disorders.

**Methods:**

This is a cross-sectional and descriptive study, carried out over a period of 4 months (February 2021-May 2021) on one hundred and twelve inmates of the civil prison of Mahdia followed in psychiatry. Data were collected using a pre-established questionnaire. It is made up of 30 items.

**Results:**

The prevalence of mental disorders in prison was 9.03%. The descriptive study revealed an average age of 37.57 years, a majority having a single marital status (62.5%), the professional activity before imprisonment were workers in 61.6%, a history of imprisonment more than twice in 62.5% of cases and 50.89% declared having been victims of physical acts, psychological or sexual abuse during their childhood. Murder, armed robbery, drug trafficking and rape were the most frequent offenses with respective rates of 25.2; 17.07; 13.82 and 9.75%. Anxiety was noted in 53.57% of cases, of the respondents, depressive syndrome was in 28.57% of cases, schizophrenia was reported in 18, 75% of cases and substance-related disorders were noted in 21.42% of cases.

**Conclusions:**

Longitudinal studies should, in the coming years, try to understand the impact of imprisonment on the onset and evolution of psychiatric disorders.

**Disclosure:**

No significant relationships.

